# Temporal trend of hepatitis B surface mutations in the post-immunization period: 9 years of surveillance (2005–2013) in eastern China

**DOI:** 10.1038/s41598-017-07085-z

**Published:** 2017-07-27

**Authors:** Bingyu Yan, Jingjing Lv, Yi Feng, Jiaye Liu, Feng Ji, Aiqiang Xu, Li Zhang

**Affiliations:** 1 Academy of Preventive Medicine, Shandong University, Jinan, China; 2 Shandong Provincial Key Laboratory of Infectious Disease Control and Prevention, Shandong Center for Disease Control and Prevention, 16992, Jingshi Road, Jinan, 250014 China

## Abstract

Limited information is available about the temporal trend in the prevalence and evolution of hepatitis B virus (HBV) *S*-gene mutations in the post-immunization era in China. From 2005 to 2013, 1077 hepatitis B cases under 15 years of age reported through Chinese National Notifiable Disease Reporting System (NNDRS) were successfully sequenced of *S*-gene in Shandong province, China. A total of 97 (9.01%) cases had amino acid (aa) substitution in the “α” determinant of HBsAg. The yearly prevalence from 2005 to 2013 maintained at a relatively stable level, and showed no significant change (*P* > 0.05). Multivariate logistic regression analysis demonstrated that the prevalence of “α” mutations was independently associated with the maternal HBsAg status (*P* < 0.05), and not with surveillance year and hepatitis B vaccination (*P* > 0.05). The hottest mutation position was aa126 (I126S/N and T126A, 29.63%), and aa 145 (G145R/A, 25.93%). Mutated residue 126 tended to occur less frequent, while that of residue 145 was more frequent with increasing year. Our data showed that there was no increase in the frequency of HBV “α” mutations over time during the post-immunization period. However, long-term vaccination might enhance the change of HBV mutational pattern, and G145 mutation was becoming dominant.

## Introduction

Hepatitis B virus (HBV) infection and HBV-related complications remains a major global public health problem^1^. An estimated 350 million population were chronically HBV infected, with 600,000 deaths annually related to HBV worldwide^2^. In most HBV endemic areas, maternal-infant vertical transmission is a major route for the HBV chronic infection (approaching 90%)^3, 4^. Interruption of early HBV transmission through effective immunization is the most cost-effective strategy for the prevention of primary infection.

The surface (S) region of HBV surface antigen (HBsAg), utilized in current recombinant hepatitis B vaccine (HepB), contains a highly conserved antibody-neutralizing epitope cluster which spans amino acids (aa) 124–147 within the major hydrophilic region (MHR) of HBsAg, and is referred to as “α” determinant. The “α” determinant is considered to be in the form of two major loops with cysteine-disulfide bonds^5^. It is well documented that neutralizing antibodies produced during natural infection, or following active or passive immunization against HBV are targeted to the conformational epitopes of the “α” determinant^6^. Hence, single or multiple mutations occurring within this region can lead to conformational changes with altered antigenicity and can allow replication of mutated HBV in vaccinated population, posing a potential threat to the long-term success of massive vaccination^7^. Such mutations are generally behaved as vaccine escape mutants (VEMs). Early investigations have revealed that VEMs were mainly occurred in the “α” determinant^8^. G145R mutation was the first identified VEMs to be described in 1990^9^, and later several other notable VEMs (I/T126S, Q129H, G130N, D144A, G145A) at different positions associated with HBV breakthrough infections have also been repeatedly documented worldwide^10, 11^. Apart from causing immune failure, VEMs are found in association with HBV occult infection^12, 13^, and also have been proven to reduce the binding of antibodies raised against wild-type small envelop protein under experimental conditions^14, 15^.

In China, HepB was recommended to infants since 1992 and was introduced into the national children immunization program in 2002, with the prevalence of HBsAg among the children under 5 years of age decreasing from 9.67% in 1992 to 0.96% in 2006^16^. With the successful control of HBV achieved by massive hepatitis B vaccination, one of the remaining concerns might focus on the issue of VEMs, which may potentially challenge the global prevention program and the eventual eradication of HBV infection^7, 17^.

Shandong province is located in the eastern part of China and covers an area of 156,700 km^2^, which is inhabited by a population of 95.79 million (2010 census data). With the implementation of national immunization program, HBsAg prevalence among children aged 1–14 years has dramatically decreased from 8% in 1992 to 1.36% in 2006^18^. Since 2005, surveillance of hepatitis B case under 15 years of age has been implemented in the province. We used the blood samples collected in this surveillance system during 2005–2013 to analyze the trend of the prevalence and pattern of HBV *S*-gene mutations in the era of massive hepatitis B vaccination, evaluating the influence of the current national universal vaccination immunization program on HBV surface mutations.

## Methods

### Study population and sample collection

The surveillance on hepatitis B cases was initiated in all 140 counties of Shandong province since 2005. The definition of hepatitis B case was based on clinical and serological criteria in the hospital^19^. After being reported through Chinese National Notifiable Disease Reporting System (NNDRS), all hepatitis B cases aged 0–14 years, including acute and chronic cases, were investigated using a standard questionnaire through interviewing with their parents by the staffs of the Center for Disease Control and Prevention (CDC) at county level. All investigators were trained at provincial CDC before the investigation. The cases’ demographic information (age, gender and ethnicity), hepatitis B vaccination history, clinical characteristics and epidemiological risk factors (maternal HBsAg status, history of blood transfusion and operation, and so on) were collected. Hepatitis B vaccination history was obtained according to immunization certificate, if not, by parents’ description. Blood samples were collected for each case in the hospitals and then transferred to the provincial CDC and stored at −70 °C until use.

The ethical approval was given by Ethics Review Committee of the Shandong CDC, and the study was conducted in accordance with the ethical standards of the Declaration of Helsinki. Written informed consents for the use of their clinical samples were obtained from the children’s legal guardians.

### HBV DNA extraction and nested polymerase chain reaction

HBV DNA was extracted from 200 ul serum with QIAamp DNA Blood Mini Kit (QIAGEN, Hilden, Germany) according to manufacturer’s instructions. DNA was amplified by two primers, HBVSF1 (sense, nucleotides [nt] 203–221: 5′-CCTGTATTTTCCTGCTGGTGGCTCC-3′) and HBVSR1 (antisense, nt 1002–1022, 5′-GCAGCAAAGCCCAAAAGACCC-3′) in a 50-UL reaction. Amplification conditions were as follows: 94 °C for 5 min, then 35 cycles at 94 °C for 35 s, 58 °C for 30 s, 72 °C for 35 s, and finally 72 °C for 10 min. Amplified products were visualized by 1.5% agarose gel electrophoresis stained with GelRed^TM^ and evaluated under UV light. Samples that were found to be negative in the one-step PCR were amplified by two-step PCR using HBVSF2 (sense, nt 196–213: 5′-GTTACAGGCGGGGTTTTT-3′) and HBVSR2 (antisense, nt 860–881, 5′-CCCATGAAGTTAAGGGAGTAGC-3′) inner primers in second round. The amplification conditions were the same as in the one-step PCR. All necessary precautions to prevent cross-contamination were strictly followed, and negative controls were included in each step of the molecular assays.

### DNA sequencing and mutation analysis

The positive PCR products were purified and sequenced directly with a BigDye Terminator v3.1 Cycle Sequencing Kit (Applied Biosystems, Foster City, CA). Sequences were analyzed by an ABI Prism 3130X genetic analyzer (Applied Biosystems). The HBV genotype was identified by phylogenetic analysis using neighbor-joining method after estimation of genetic distance using the Kimura two-parameter method (Mega, v4.0)^20^, including 28 HBV sequences of different genotypes (A-H) obtained from GenBank. The reliability of the phylogenetic tree was tested using the bootstrap test with 1,000 replicates. The HBV serotype was deduced from the amino acid (aa) at positions 122, 127, 134, 159, and 160^21^. The envelope gene aa sequence of each sample was determined by translation of the nucleotide sequence according to the S open reading frame (ORF). Amino acid mutations were identified by comparison of the target sequence with HBV reference sequences of the same genotype. The sequences obtained were submitted to GenBank with the assessions KX067076-KX067778.

### Statistical analysis

Comparison of categorical variables among different groups was analyzed using *χ* ^2^ test or Fisher’s exact test, as appropriate. The *χ* ^2^ test for trend was used to analyze the trend of proportions with year. A logistic regression model was used to examine associations between the prevalence of “α” mutations and putative risk factors. All statistical tests were two sided. *P* value < 0.05 was considered statistically significant. All statistical analysis was performed by SPSS 13.0 for Windows (SPSS Inc., IL).

## Results

### HBV DNA profile

**Figure 1:**
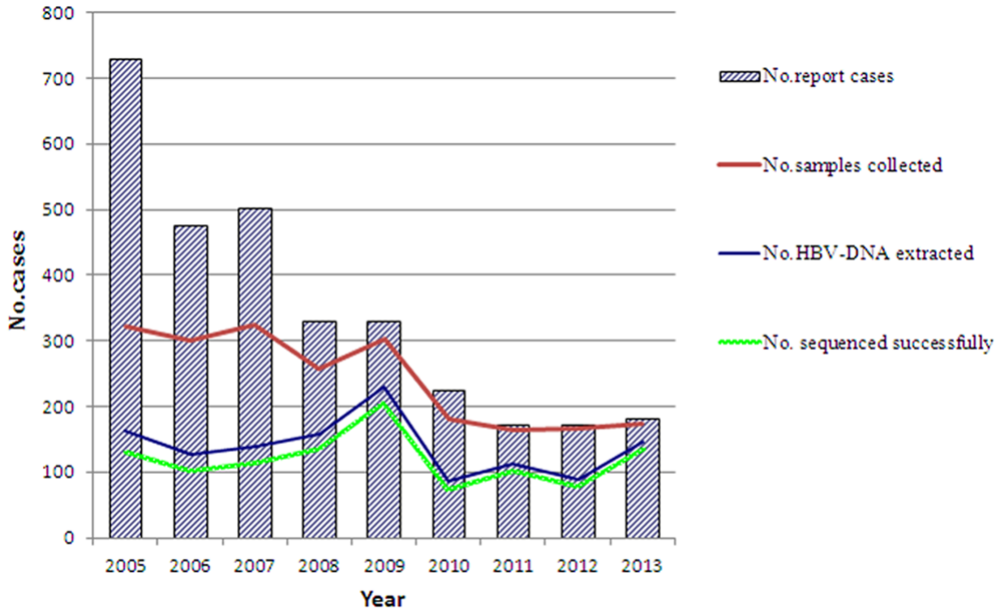
Annual numbers of HBV cases aged 0–14 years reported through NNDRS, with samples collected, HBV-DNA extracted and successfully sequenced in Shandong Province, China, 2005–2013.

From 2005 to 2013, a total of 3120 cases of hepatitis B under15 years of age (average age: 9.4 ± 4.2 years, male/female ratio: 2.05/1) were reported by NNDRS in Shandong province, China. The case number decreased with year during this period. Blood samples were collected from 2194 (70.32%) of them. Due to an insufficient volume of serum, HBV-DNA extraction was completed only for 1248 (56.88%, 1248/2194) cases, with S gene successfully sequenced in 1077 cases (average age: 9.4 ± 4.5 years, male/female ratio: 2.49/1) (Fig. 1).

### Genotypes and serotypes of HBV strains

Phylogenetic analysis of S gene indicated that three HBV genotypes were circulating in Shandong province. Genotype C was predominant, accounting for 93.59% (1008/1077); followed by genotype B for 6.13% (66/1077) and genotype D for 0.28% (3/1077). No other genotypes were detected. The majority of HBV strains belonged to HBsAg subtype *Adrq* ^+^ (88.67%, 955/1077) based on Lys122, Arg160, Val177 and Pro178; four strains (0.37%) were specified as *Adrq* ^−^, based on Lys122, Arg160 and Ala177; ninety-six strains (8.91%) belonged to *adw2*, based on Lys122, Lys160 and Pro127; three strains (0.29%) were specified as *adw3*, based on Lys 122, Lys160 and Thr127; thirteen strains (1.21%) belonged to *ayr*, based on Arg122 and Arg 160; six strains (0.56%) belonged to *ayw1*, based on Arg122, Lys160, Pro127, Phe134 and/or Ala159.

### Prevalence of “α” determinant mutations

In 1077 acquired sequences, a total of 97 (9.01%, 97/1077) strains had aa substitution in the “α” determinant region of HBsAg. The yearly prevalence of “α” mutations during 2005–2013 was 6.11% (8/131), 9.90% (10/101), 9.73% (11/113), 6.62% (9/136), 9.22% (19/206), 9.33% (7/75), 12.75% (13/102), 10.26% (8/78), and 8.89% (12/135), respectively, showing no statistically significant difference.

To further investigate the profiles of “α” determinant mutations, 403 cases with missing information on hepatitis B vaccination and/or maternal HBsAg status were excluded. Table 1 listed the virological characteristics of the remaining 674 cases with the stratification by the vaccination status and maternal HBsAg. Surveillance year, cases’ age, gender, HBeAg status, hepatitis B vaccination, maternal HBsAg status, and HBV genotypes were included in the multivariate regression model (Table 2). It was found that maternal positive-HBsAg was a risk factor independently associated with the occurrence of “α” determinant mutations, while the other factors including cases’ age, gender, HBeAg status, hepatitis B vaccination and HBV genotype were not associated.

**Table 1:** Prevalence of “α” determinant mutations among hepatitis B cases according to selected characteristics.

Variable	Vaccinated						Unvaccinated					
	Maternal HBsAg +		Maternal HBsAg (−)		Total		Maternal HBsAg+		Maternal HBsAg (−)		Total	
	No.	Mutants (n,%)	No.	Mutants (n,%)	No.	Mutants (n,%)	No.	Mutants (n,%)	No.	Mutants (n,%)	No.	Mutants (n,%)
Total	245	27 (11.02)	271	18 (6.64)	516	45 (8.72)	45	5 (11.11)	113	7 (6.19)	158	12 (7.59)
Surveillance year												
2005–2007	50	9 (18.00)	77	3 (3.90)	127	12 (9.45)	17	0 (0.00)	60	6 (10.00)	77	6 (7.79)
2008–2010	100	11 (11.00)	97	5 (5.15)	197	16 (8.12)	19	4 (21.05)	39	1 (2.56)	58	5 (8.62)
2011–2013	95	7 (7.37)	97	10 (10.31)	192	17 (8.85)	9	1 (11.11)	14	0 (0.00)	23	1 (4.35)
Age (yrs)												
0–4	78	9 (11.54)	53	5 (9.43)	131	14 (10.69)	10	1 (10.00)	4	0 (0.00)	14	1 (7.14)
5–9	78	10 (12.82)	53	6 (11.32)	131	16 (12.21)	8	1 (12.50)	20	1 (5.00)	28	2 (7.14)
10–14	89	8 (8.99)	165	7 (4.24)	254	15 (5.91)	27	3 (11.11)	89	6 (6.74)	116	9 (7.76)
Birth cohort												
Before 2002	119	13 (10.92)	184	10 (5.43)	303	23 (7.59)	31	3 (9.68)	104	7 (6.73)	135	10 (7.41)
2002–2006	70	10 (14.29)	50	2 (4.00)	120	12 (10.00)	6	1 (16.67)	8	0 (0.00)	14	1 (7.14)
After 2006	56	4 (7.14)	37	6 (16.22)	93	10 (10.75)	8	1 (12.50)	1	0 (0.00)	9	1 (11.11)
Gender												
Male	166	17 (10.24)	198	10 (5.05)	364	27 (7.42)	27	4 (14.81)	89	7 (7.87)	116	11 (9.48)
Female	79	10 (12.66)	73	8 (10.96)	152	18 (11.84)	18	1 (5.56)	24	0 (0.00)	42	1 (2.38)
Children’s HBeAg^a^												
Positive	175	18 (10.29)	146	8 (5.48)	321	26 (8.10)	36	4 (11.11)	79	3 (3.80)	115	7 (6.09)
Negative	31	6 (19.35)	60	3 (5.00)	91	9 (9.89)	4	1 (25.00)	17	2 (11.76)	21	3 (14.29)
Unknown	39	3 (7.69)	65	7 (10.77)	104	10 (9.62)	5	0 (0.00)	17	2 (11.76)	22	2 (9.09)
Genotype												
B	11	2 (18.18)	22	3 (13.64)	33	5 (15.15)	2	1 (50.00)	6	0 (0.00)	8	1 (12.50)
C	234	25 (10.68)	249	15 (6.02)	483	40 (8.28)	43	4 (9.30)	107	7 (6.54)	150	11 (7.33)

^a^Children’s HBeAg was obtained from the medical records in hospital.

**Table 2:** Factors associated with the occurrence of “α” determinant mutations in multivariate logistic regression among hepatitis B cases aged 0–14 years.

Variables	Category	*P*	OR^a^	95%CI^b^
Surveillance year	2005–2007		1.00	
	2008–2010	0.583	0.83	0.42–1.68
	2011–2013	0.606	0.83	0.40–1.70
Age (yrs)	0–4		1.00	
	5–9	0.728	1.14	0.54–2.41
	10–14	0.352	0.71	0.34–1.46
Gender	Female		1.00	
	Male	0.649	0.87	0.48–1.57
Hepatitis B vaccination	No		1.00	
	Yes	0.819	0.92	0.45–1.87
Maternal HBsAg	Negative		1.00	
	Positive	0.046	1.82	1.01–3.26
Children’s HBeAg	Negative		1.00	
	Positive	0.144	0.58	0.28–1.20
Genotype	B		1.00	
	C	0.154	0.50	0.20–1.29

^a^Odds ratio (OR) and ^b^95% confidence interval (95% CI) for OR were calculated to assess the association between the occurrence of “α” determinant mutations and relevant factors. The statistical significance level of *P* < 0.05 was used.

Fifty-seven out of 674 cases with complete information had “α” mutations. Documented anti-HBs titer and HBV DNA levels were available in 42 cases among them. Of these, five cases (No. 11, 24, 29, 33, 43) had a protective level of anti-HBs (138.8, 182, 120, 21.78 and 123.38 mIU/ml, respectively). Quantification of HBV DNA revealed levels ranging from <500 to 3.84 × 10^8^ IU/ml. The mutations cases’ characteristics were summarized in Supplement-1.

### Mutation type of the “α” determinant domain in HBV isolates

Among 88 mutated isolates with HBV genotype C, a total of 98 aa substitutions with sixteen mutational types at 10 aa sites occurred, including 80 isolates with a single mutation, 6 isolates with double mutations and 2 isolates with triple mutations. Eight types of these mutations (I126S, I126N, Q129H, S143L, D144A, D144E, G145A, and G145R) had demonstrated low ability to bind antibodies and were identified as VEMs previously^9, 22–27^. Notably, two T131N/M133T (a novel N-linked glycosylation site) double mutations associated with I126S were observed in our study^15^. In addition, the other six mutations types (P127T, A128V, G130E, T131P, M133I and D144N) were not yet identified as VEMs, and D144N was a newly identified mutation (Table 3). Among 9 mutated isolates sharing 6 types of aa substitutions with HBV genotype B, three types of VEMs (T126A, Q129H and D144A) were also detected (Table 4).

**Table 3:** Amino acid mutations in the “α” determinant of HBsAg from genotype C among HBV-infected children.

Amino acid mutations^a^	Frequency^b^	Percentage (%)	Reported function	Reference^c^
I126S	24	24.49	**Vaccine escape mutant**	22
I126N	6	6.12	**Vaccine escape mutant**	22
P127T	10	10.2	Unclear	41
A128V	1	1.02	lower reactivity in HBsAg assay	13, 42
Q129H	3	3.06	**Vaccine escape mutant**	23
G130E	1	1.02	lower reactivity in HBsAg assay	13
T131P	7	7.14	Unclear	14
T131N	4	4.08	Rescue of virion secretion	15
M133T	4	4.08	Rescue of virion secretion	15
M133I	2	2.04	Unclear	22
S143L	1	1.02	**Vaccine escape mutant**	24
D144A	3	3.06	**Vaccine escape mutant**	25
D144E	3	3.06	**Vaccine escape mutant**	26
D144N	1	1.02	Unclear	Novel
G145A	23	23.47	**Vaccine escape mutant**	27
G145R	5	5.1	**Vaccine escape mutant**	9

^a^Amino acid aa mutants were presented based on their location in the archived GenBank reference AF473543. ^b^Frequency reflected the number of cases in which the mutations was detected. ^c^Unreported mutants were considered novel.

**Table 4:** Amino acid mutations in the “α” determinant of HBsAg from genotype B among HBV-infected children.

Amino acid mutations^a^	Frequency^b^	Percentage (%)	Reported function	Reference
T126A	2	20.00	Vaccine escape mutant	22
P127T	3	30.00	Unclear	41
Q129H	2	20.00	Vaccine escape mutant	23
M133T	1	10.00	Rescue of virion secretion	15
F134L	1	10.00	lower reactivity in HBsAg assay	13
D144A	1	10.00	Vaccine escape mutant	25

^a^Amino acid aa mutants were presented based on their location in the archived GenBank reference FJ562246. ^b^Frequency reflected the number of cases in which the mutations was detected.

The most prevalent mutation position was aa 126 [I126S (n = 24), I126N (n = 6), T126A (n = 2), 29.63%], and aa145 [G145R (n = 5), G145A (n = 23), 25.93%], all of which were recognized as VEMs^9, 22, 27^. Seventy-one (65.74%, 71/108) out of 107 mutations were located in the 1st loop (aa 124–137) and 37 (34.26%, 37/108) in the 2nd loop (aa 139–147) of this antigenic domain.

**Figure 2:**
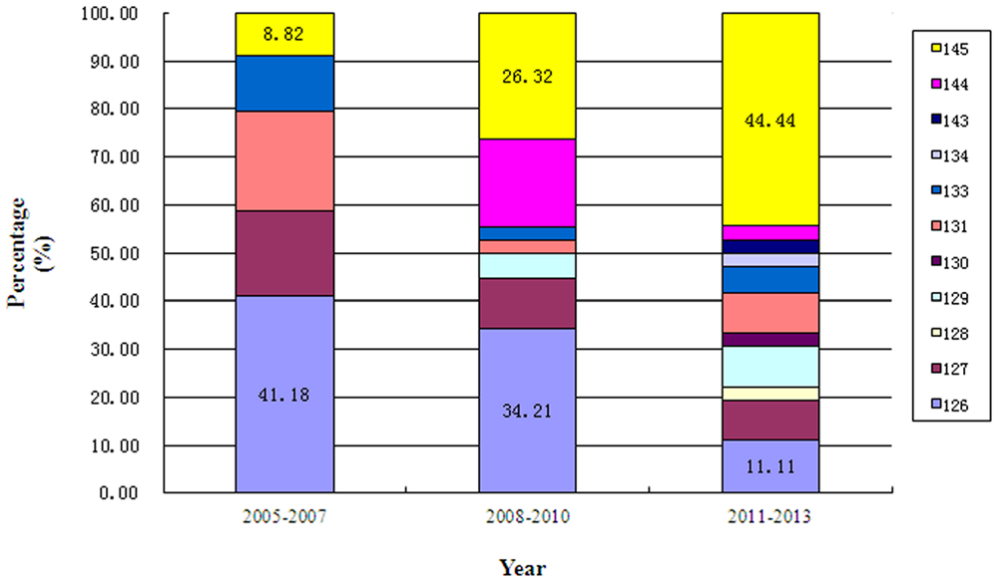
Percentage of different amino acid position mutations within “α” determinant of HBsAg from 2005 to 2013.

Interestingly, a significant change regarding to the mutation frequency of aa 126 and aa 145 was observed from 2005 to 2013. The percentage of mutation position at aa 126 was 41.18%, 34.21% and 11.11% in the year of 2005–2007, 2008–2010 and 2011–2013, showing a significantly downward trend with year (*χ* ^2^_trend_ = 7.74, *P* = 0.005). By contrast, the percentage of aa 145 was 8.82%, 26.32% and 44.44% in the corresponding period, showing a significantly upward trend (*χ* ^2^_trend = _11.20, *P* = 0.001) (Fig. 2).

## Discussion

A nationwide seroepidemiologic survey in China showed that a hepatitis B vaccination coverage rate (3 doses of vaccine) increased from 60.8% for children born in 1992–1997 to 93.2% in 2002–2005^28^, and now hepatitis B vaccination coverage has expanded to almost all children in China. Our finding revealed that the prevalence of “α” mutations maintained at a relatively low and stable level, and showed no significant change during the consecutive 9-year of the massive vaccine period, similar to a previous report in Taiwan^29^. Multivariate logistic regression analysis also revealed that the prevalence of “α” mutations was not associated with the surveillance year, indicating that the universal infant immunization did not obviously accelerate the frequency of HBV “α” mutations over time in the post-immunization era, and also suggested a low transmissibility of the mutational viruses. However, it has been reported recently that early universal immunization might accelerate the “α” mutations in vaccinated children^30, 31^. Due to lack of the baseline data during pre-vaccination period in Shandong province, our study could not compare the epidemiological difference of “α” mutations before and after the massive vaccination. Further, our study showed no difference between the children with and without immunization, like the findings in four Pacific Island countries^32^, and in Thailand^33^. It is possible that the frequency of “α” mutations was not tightly related to the single vaccination, and most of these mutations might emerge spontaneously. Because of the small sample size of unvaccinated children in the study, it is essential to conduct a further investigation with increasing sample size.

The present study found a much higher prevalence of “α” mutations among children with HBsAg-positive mother compared with those whose mother was HBsAg-negative. The reason might be the fact that most children born to HBsAg-positive mother were given additional hepatitis B immunoglobulin (HBIG) besides three doses of hepatitis B vaccine to interrupt HBV vertical transmission^34^, which was reported to be a major driving force for the selection of VEMs^7^.

Based on an alarming number of sequences obtained in the present study, several important findings were observed concerning the mutation patterns. Of the 11 positions where mutations occurred in “α” determinant region, the most frequent mutation position was aa 126 (I126S/N and T126A, 29.63%), and 145 (G145R/A, 25.93%), all of which were known to act as VEMs, accounting for more than half of the total mutation strains wither the “α” determinant. So, aa 126 and 145 might be the hottest mutation positions among HBV-infected children. Residue 126 is located in the 1st loop structure of the “α” determinant, and aa changes in this region may lead to HBV adaptive evolution, resulting in the changes of antigenicity and virulence increased^26, 35^. Mutations at aa 126 had been found frequently in HBV isolates from patients with chronic HBV infection with or without immunization, and most of these mutations were considered to be natural occurrence^10^. Residue 145 is located in the 2nd loop structure, known to be the best-described escape mutant position. G145 mutation is highly associated with vaccination selection, and neutralizing antibody induced by vaccination is no longer recognizes the mutated epitope^9, 27^. The well-known G145R/A mutations were often found in vaccination failure settings^10^. Again, an *in vitro* model revealed that HBV with a G145R mutation shared the replication efficacy with equal to wild-type virus^36^.

Our study found mutated residue 126 tended to occur less frequent, while that of aa 145 was more frequent with increasing year. It was largely attributable to an increasing massive vaccination and favoring selective pressure on HBV, and accordingly indicating that the substitutions in the aa positions 145 were more associated with anti-HBs immune pressure than those in other epitope sites of “α” determinant region. Our results strongly suggested although the prevalence of “α” determinant mutations showed no significant change in the post-immunization era, the patterns of these mutations had been remarkably changed under the pressure of vaccination. As a result, G145 mutation was becoming more and more dominant with the vaccination selection and needed to be incorporated into future vaccine design.

Four other types of VEMs (Q129H, S143L, D144A, D144E), which were known to be associated with virus secretion and lower reactivity in HBsAg assays, were also detected^23–26^. Additionally, a small amount of P127T, A128V, G130E, T131P/N, M133T/I, F134L and D144N mutations was observed in our study, including the less frequent T131N/M133T double mutations. T131N/M133T can create a novel N-linked glycosylation site, and play a role in some HBV immune escape mutants^15^. Further biological functional analyses of these mutations are needed to determine whether they are able to escape existing vaccines. To the best our knowledge, D144N is a new identified mutation in the present study.

So far, it is still uncertain when these mutations emerged, or their duration in circulation. A previous study proved that more than half of HBV-infected children could spontaneously lose the predominance of circulatory mutant virus by reversion to wild-type^37^. However, Hsu *et al*. confirmed that G145R virus could circulate as a stable strain for 8 years of follow-up^38^. Such mutations could also contribute to HBV transmission in children born to HBsAg-positive mothers and vaccinated individuals^39, 40^.

There were still several limitations in our study. The data related to the administration of HBIG backgrounds in the studied children was not been collected. No children with HBV occult infection were involved in our study, who were reported to harbor even more mutations^12^. The point mutations of “α” determinant were detected by direct sequencing instead of cloning and sequencing assay and quasispecies mutations might be ignored in this study, which led to the underestimation of actual prevalence of “α” determinant mutations.

In conclusion, the present study revealed that the prevalence of “α” determinant mutations remained stable in post-immunization period. However, long-term hepatitis B vaccination enhances the change of epidemic types in “α” determinant mutations. G145 mutation was becoming dominant. Given the facts that hepatitis B vaccine, HBIG and antiviral drug would more and more widely used in the future and possibility of the transmission of such “α” determinant mutation has not be complete ruled out, an ongoing surveillance with longer years is desperately needed.

## Electronic supplementary material

Supplementary Information

## References

[1] Schweitzer A, Horn J, Mikolajczyk RT, Krause G, Ott JJ (2015). Estimations of worldwide prevalence of chronic hepatitis B virus infection: a systematic review of data published between 1965 and 2013. Lancet..

[2] Perz JF, Armstrong GL, Farrington LA, Hutin YJ, Bell BP (2006). The contributions of hepatitis B virus and hepatitis C virus infections to cirrhosis and primary liver cancer worldwide. J Hepatol..

[3] Broderick AL, Jonas MM (2003). Hepatitis B in children. Semin Liver Dis.

[4] Compri AP (2012). Hepatitis B virus infection in children, adolescents, and their relatives: genotype distribution and precore and core gene mutations. Rev Soc Bras Med Trop.

[5] Coleman PF (2006). Detecting hepatitis B surface antigen mutants. Emerg Infect Dis.

[6] Zuckerman JN, Zuckerman AJ (2003). Mutants of the surface protein of hepatitis B virus. Antiviral Res.

[7] Romanò L (2015). Hepatitis B vaccination. Hum Vaccin Immunother.

[8] Pollicino T, Cacciola I, Saffioti F, Raimondo G (2014). Hepatitis B virus PreS/S gene variants: pathobiology and clinical implications. J Hepatol.

[9] Carman WF (1990). Vaccine-induced escape mutant of hepatitis B virus. Lancet.

[10] Cooreman MP, Leroux-Roels G, Paulij WP (2001). Vaccine and hepatitis B immune globulin-induced escape mutations of hepatitis B virus surface antigen. J Biomed Sci.

[11] Theamboonlers A, Chongsrisawat V, Jantaradsamee P, Poovorawan Y (2001). Variants within the “a” determinant of HBsAg in children and adolescentsn with and without hepatitis B vaccination as part of Thailand’s Expanded Program on Immunization (EPI). Tohoku J Exp Med.

[12] Raimondo G, Pollicino T, Romanò L, Zanetti AR (2010). A 2010 update on occult hepatitis B infection. Pathol Biol (Paris).

[13] Yong-Lin Y (2012). Hepatitis B surface antigen variants in voluntary blood donors in Nanjing, China. Virol J.

[14] Huang CH (2012). Influence of mutations in hepatitis B virus surface protein on viral antigenicity and phenotype in occult HBV strains from blood donors. J Hepatol.

[15] Kwei K (2013). Impaired virion secretion by hepatitis B virus immune escape mutants and its rescue by wild-type envelope proteins or a second-site mutation. J Virol.

[16] Liang X (2009). Evaluation of the impact of hepatitis B vaccination among children born during 1992–2005 in China. J Infect Dis.

[17] Hsu HY (2015). Universal infant immunization and occult hepatitis B virus infection in children and adolescents: a population-based study. Hepatology.

[18] Zhang L (2010). A significant reduction in hepatitis B virus infection among the children of Shandong Province, China: the effect of 15 years of universal infant hepatitis B vaccination. Int J Infect Dis.

[19] Chinese Society of Hepatology, Chinese Medical Association, Chinese Society of Infectious Diseases. & Chinese Medical Association. The guidelines of prevention and treatment for chronic hepatitis B. *Zhonghua Gan Zang Bing Za Zhi* **13**, 881–91 Chinese (2005). 16491521

[20] Tamura K, Dudley J, Nei M, Kumar S (2007). MEGA4: Molecular evolutionary genetics analysis (MEGA) software version 4.0. Mol. Biol. Evol.

[21] Magnius LO, Norder H (1995). Subtypes, genotypes and molecular epidenmiology of the hepatitis B Virus as reflected by sequence variability of the S-gene. Intervirology.

[22] He C, Nomura F, Itoga S, Isobe K, Nakai T (2001). Prevalence of vaccine-induced escape mutants of hepatitis B virus in the adult population in China: A prospective study in 176 restaurant employees. J Gastroenterol Hepatol.

[23] Carman WF (1997). The clinical significance of surface antigen variants of hepatitis B virus. J Viral Hepat.

[24] Wang XY (2015). The prevalence of mutations in the major hydrophilic region of the surface antigen of hepatitis B virus varies with subgenotype. Epidemiol Infect.

[25] Jolivet-Reynaud C (2001). Localization of hepatitis B surface antigen epitopes present on variants and specifically recognised by anti-hepatitis B surface antigen monoclonal antibodies. J Med Virol.

[26] Torresi J (2002). Reduced antigenicity of the hepatitis B virus HBsAg protein arising as a consequence of sequence changes in the overlapping polymerase gene that are selected by lamivudine therapy. Virology.

[27] Seddigh-Tonekaboni S (2001). Hepatitis B surface antigen variants in vaccines, blood donors and an interferon-treated patient. J Viral Hepat.

[28] Cui F (2010). Factors associated with effectiveness of the first dose of hepatitis B vaccine in China: 1992-2005. Vaccine.

[29] Hsu HY (2010). No increase in prevalence of hepatitis B surface antigen mutant in a population of children and adolescents who were fully covered by universal infant immunization. J Infect Dis.

[30] Bian T (2013). Change in hepatitis B virus large surface antigen variant prevalence 13 years after implementation of a universal vaccination program in China. J Virol.

[31] Hsu HY, Chang MH, Liaw SH, Ni YH, Chen HI (1999). Changes of hepatitis B surface variants in carrier children before and after universal vaccination in Taiwan. Hepatology.

[32] Basuni AA, Butterworth L, Cooksley G, Locarnini S, Carman WF (2004). Prevalence of HBsAg mutants and impact of hepatitis B infant immunization in four Pacific Island countries. Vaccine.

[33] Yimnoi P (2016). A molecular epidemiological study of the hepatitis B virus in Thailand after 22 years of universal immunization. J Med Virol.

[34] Zhang L (2014). Perinatal hepatitis B prevention program in Shandong Province, China. Evaluation and progress. Hum Vaccin Immunother.

[35] Qiu S (2008). Reduced Antigenicity of Naturally Occurring Hepatitis B Surface Antigen Variants with Substitutions at the Amino Acid Residue 126. Intervirology.

[36] Jammeh S, Thomas HC (2007). Replicative competence of the T131I, K141E, and G145R surface variants of hepatitis B Virus. J Infect Dis.

[37] Hsu HY (2013). Long-term follow-up of children with postnatal immunoprophylaxis failure who were infected with hepatitis B virus surface antigen gene mutant. J Infect Dis.

[38] Hsu HY (1997). Surface gene mutants of hepatitis B virus in infants who develop acute or chronic infections despite immunoprophylaxis. Hepatology.

[39] Shahmoradi S (2012). High prevalence of occult hepatitis B virus infection in children born to HBsAg-positive mothers despite prophylaxis with hepatitis B vaccination and HBIG. J Hepatol.

[40] Lai MW (2012). Increased seroprevalence of HBV DNA with mutations in the s gene among individuals greater than 18 years old after complete vaccination. Gastroenterology.

[41] Mele A (2001). Effectiveness of hepatitis B vaccination in babies born to hepatitis B surface antigen-positive mothers in Italy. J Infect Dis.

[42] Katsoulidou A (2009). Molecular characterization of occult hepatitis B cases in Greek blood donors. J Med Virol.

