# Supplementary material for: Temporal trend of hepatitis B surface mutations in the post-immunization period: 9 years of surveillance (2005–2013) in eastern China
Source: Sci Rep. 2017 Jul 27;7:6669. doi: 10.1038/s41598-017-07085-z (PMC5532365; doi:10.1038/s41598-017-07085-z)
Supplement: Supplementary file 1 — Supplementary Information [file 41598_2017_7085_MOESM1_ESM.pdf]

Temporal trend of hepatitis B surface mutations in the post-immunization period: 9 years of surveillance (2005- 2013) in eastern China

Bingyu Yan, Jingjing Lv, Yi Feng, Jiaye Liu, Feng Ji, Aiqiang Xu& Li Zhang

Case No.	Surveillance year	Age(yrs)	Gender	HBeAg[a]	Materal HBsAg	HepB[b]	Genotype	Viral load(IU/ml)	Anti-HBs(mIU/ml)	Amino acid substitution
1	2005	14	M	ND	+	Y	C	1.70×10^{6}	<10	I126S
2	2005	3	M	+	+	Y	C	1.64×10^{7}	<10	G145A
3	2005	14	M	(-)	(−)	N	C	7.54×10^{3}	<10	I126S+T131N+M133T
4	2005	5	M	+	+	Y	C	6.15×10^{7}	<10	T131P
5	2005	14	M	+	(−)	N	C	ND	ND	M133T
6	2006	6	M	+	+	Y	C	3.66×10^{7}	<10	P127T
7	2006	7	M	(-)	+	Y	C	4.85×10^{5}	<10	I126S
8	2006	14	M	+	(−)	N	C	2.12×10^{7}	<10	P127T
9	2006	11	F	ND	(−)	Y	C	ND	ND	T131P
10	2006	8	F	+	+	Y	C	2.56×10^{4}	<10	I126S
11	2006	10	F	+	+	Y	C	7.43×10^{4}	138.8	I126S
12	2006	14	M	+	(−)	N	C	ND	ND	I126S
13	2006	14	M	ND	(−)	N	C	2.88×10^{7}	<10	T131P
14	2006	7	M	ND	(−)	N	C	3.65×10^{7}	<10	T131P
15	2007	11	M	(-)	(−)	Y	C	ND	ND	I126S
16	2007	10	M	+	(−)	Y	C	5.26×10^{6}	<10	I126S+T131N+M133T

17	2007	7	M	(-)	+	Y	C	ND	ND	G145A
18	2007	9	F	+	+	Y	C	ND	ND	I126S
19	2008	8	F	+	(−)	Y	C	4.03×10^{7}	<10	I126S
20	2008	14	F	(-)	+	N	C	3.08×10^{6}	<10	D144E
21	2008	4	M	+	+	Y	C	8.97×10^{6}	<10	G145A
22	2008	12	F	(-)	+	Y	C	ND	ND	G145A
23	2008	13	M	(-)	+	Y	C	ND	ND	G145A
24	2009	4	F	(-)	+	Y	B	$3.14E\times10^{6}$	182.98	P127T
25	2009	3	M	+	+	Y	C	2.48×10^{6}	<10	Q129H
26	2009	11	M	+	+	N	C	2.85×10^{8}	<10	D144A
27	2009	8	M	+	(−)	Y	C	1.77×10^{3}	<10	D144N+G145R
28	2009	14	M	+	+	Y	C	4.48×10^{7}	<10	I126S
29	2009	3	F	+	+	Y	C	ND	120	M133I+D144A
30	2009	13	M	+	+	N	C	ND	ND	P127T
31	2009	1	M	(-)	+	Y	C	$<5\times10^{2}$	<10	I126S
32	2009	9	M	+	(−)	Y	C	6.23×10^{7}	<10	I126N
33	2009	2	F	ND	(−)	Y	B	1.94×10^{7}	21.78	Q129H
34	2009	14	F	ND	(−)	Y	C	ND	ND	I126S
35	2009	11	F	+	+	Y	C	1.58×10^{7}	<10	G145A
36	2010	5	M	+	+	N	C	1.73×10^{4}	<10	D144E
37	2010	8	F	+	+	Y	C	6.02×10^{7}	<10	G145R
38	2010	5	M	+	+	Y	C	6.88×10^{5}	<10	I126N+P127T
39	2010	13	M	(-)	(−)	N	C	ND	ND	I126S
40	2011	13	M	ND	+	Y	C	5.38×10^{7}	<10	G145A
41	2011	14	M	(-)	(−)	Y	C	$3.84E\times10^{8}$	<10	P127T

42	2011	6	F	ND	+	Y	C	9.83×10^{4}	<10	I126S
43	2011	2	F	+	(−)	Y	B	5.94×10^{5}	123.38	D144A
44	2011	4	M	+	+	N	B	3.01×10^{7}	<10	T126A
45	2011	5	M	+	(−)	Y	C	ND	<10	P127T
46	2011	3	M	ND	(−)	Y	B	ND	ND	Q129H
47	2012	3	M	+	+	Y	B	2.45×10^{8}	<10	F134L
48	2012	2	F	+	(−)	Y	C	$<5\times10^{2}$	<10	G145R
49	2012	8	F	ND	(−)	Y	C	1.54×10^{3}	<10	T131N
50	2012	3	M	+	+	Y	C	$<5\times10^{2}$	<10	G145A
51	2013	5	F	ND	(−)	Y	C	5.76×10^{6}	<10	G145A
52	2013	11	F	+	+	Y	C	ND	ND	G145A
53	2013	14	M	ND	(−)	Y	C	1.43×10^{3}	<10	I126S+G130E
54	2013	1	M	(-)	(−)	Y	C	5.90×10^{7}	<10	G145A
55	2013	4	M	+	+	Y	C	1.02×10^{8}	<10	G145A
56	2013	12	M	+	(−)	Y	C	$<5\times10^{2}$	<10	G145A
57	2013	8	M	+	+	Y	C	1.40×10^{8}	<10	G145A

Table . Demographic, serologic, virologic characteristics and genomic variability of HBV in 57 hepatitis B cases with harbored “α” determinant mutations. [a]: ND: no data available; [b]:Y: Hepatits B vaccinated; N: Hepatits B unvaccinated
